# Deficit sustainability and fiscal theory of price level: the case of Italy, 1861–2020

**DOI:** 10.1007/s10663-023-09577-w

**Published:** 2023-05-17

**Authors:** Emilio Congregado, Carmen Díaz-Roldán, Vicente Esteve

**Affiliations:** 1 Department of Economics & CCTH, Universidad de Huelva, Huelva, Spain; 2 Universidad de Castilla-La Mancha, La Mancha, Spain; 3 Departamento de Economia Aplicada II, Universidad de Valencia, Avda. dels Tarongers, s/n, 46022 Valencia, Spain; 4 Universidad de Alcalá, Madrid, Spain

**Keywords:** Fiscal theory of the price level, Monetary and fiscal dominance, Fiscal sustainability, Inflation, Public debt, Explosiveness, Cointegration, Multiple structural breaks, E62, H62, O52

## Abstract

We test sustainability of the Italian government deficit over the period 1861–2020 using the fiscal theory of the price level (FTPL). This approach takes into account monetary and fiscal policy interactions and assumes that fiscal policy may determine the price level even if monetary authorities pursue an inflation-targeting strategy. We consider a cointegrated model with multiple structural changes to characterize the sustainability of public finances and the prevalence of monetary or fiscal dominance during subperiods. We also use recursive unit root tests for explosiveness to test fiscal sustainability and to detect episodes of potential explosive behaviour in Italian public debt. We find two structural changes for the public debt and one change in the primary budget surplus, the alternation of monetary and fiscal dominant regimes, as well as evidence of bubbles related to three episodes of the Italian fiscal performance. Our results reveal the sensitiveness of the primary balance and the debt paths to shocks hitting fiscal, macroeconomic, and financial variables.

## Introduction

Questions such as the balancing of budget deficits, the interactions between monetary and fiscal policies, and the fiscal discipline required in monetary unions, have also been intensively discussed over the last decades. In particular, one of the main problems concerning fiscal authorities is the sustainability of government deficits, which is related to the issue of long-run solvency.

The recent international economic crisis, triggered by COVID-19 pandemic, and the attempt to alleviate it through Keynesian policies has put public budget figures in the red and it has turned the attention of governments back to the crucial issue of fiscal sustainability. The ways to deal with the crisis have shown that the role of fiscal policy goes beyond the traditional stabilization function.

The question that arises is whether the current fiscal policy measures would be sustainable in the next future or, in the contrary, those fiscal packages would lead to an unsustainable path of deficit and debt.

Fiscal policy is regarded as sustainable when, if maintained in the indefinite future, it does not violate the solvency constraint; and a government is said to be solvent if the present value budget constraint, i.e., its intertemporal budget constraint (IBC) holds. In other words, the public deficit can be sustainable if the government can borrow. However, if the interest rate on the government debt exceeds the growth rate of the economy, debt dynamics would lead to an ever-increasing ratio of debt to GDP. The dynamics of debt accumulation can only be stopped only if the ratio of the budget deficit to GDP would turn to be a surplus, or if seigniorage were allowed for.

To address the above issues, the case of Italy is paradigmatic. The Italian case proves to be of special interest given the permanent difficulties experienced when balancing the government budget across years, and it is also an interesting case study among eurozone countries. Given that the Italian fiscal performance has been characterized by chronic government deficits and episodes with high levels of public debt, which is particularly dangerous when belonging to a monetary union.

Since the start of the COVID-19 crisis, Italian government has focused on doing whatever it takes to limit its social and economic consequences. The response of Italian fiscal policy for an unprecedented crisis has included, among others: a) public health measures; b) deferral and suspension of taxes on businesses (the self-employed hit hardest by the pandemic exempted from paying social security); c) subsidisation of labour costs and unemployment (partial unemployment arrangements as to the employer exemption from paying the usual labour subsidy of "cassa integratione" scheme; the ban on dismissals to workers subject to the "cassa integrazione" scheme), and others several support for businesses (hospitality sector, discounts on electricity expenditure, non-refundable contributions to the VAT item, and special aid fund to support the winter tourism industry, among others); d) public guarantees, liquidity measures and firms’ capitalisation (large firms and SMEs); e) support for households (small aid for the purchase of various consumer goods, aid for paying rent and utility bills, increased appropriations to several funds to combat poverty, actions to encourage remote work; and f) job retention schemes, for both paid employees and self-employed workers.

The massive fiscal support, provided since the start of the COVID-19 crisis, has succeeded in protecting people and preserving jobs. But it has considerably increased public expenditure and, together with sharp falls in tax revenues owing to the recession, it has pushed the Italian public debt to a recent all-time high of 155.8 percent of GDP in 2020, but is projected to fall to 146.8% by 2023, thanks also to GDP growth. In the eurozone (as in other advanced economies and some emerging market economies), European Central Bank purchases of government debt have helped to keep interest rates at historic lows and have supported government borrowing.

In this paper, we provide a formal test of the sustainability of the Italian government deficit over the period 1861–2020. In an attempt to disentangle the implications of the Italian deficit on the interactions between fiscal and monetary policies, we have also analyzed the role played by monetary and fiscal dominance in order to get fiscal solvency along the period. The nature of that relationship would inform us about the dynamics of the successive Italian goverment’s macroeconomic performance along the time.

Several studies have dealt with the issue of fiscal dominance in Italian economy using a short or a long sample, but no clear evidence on the prevalence of a "monetary dominant" (MD) or a "fiscal dominant" (FD) regime was found. However, this type of evidence is not conclusive and it is not robust to the time span, as well to the estimation methodology. The use of a longer than usual span of data (i.e., 160 years) should allow us to obtain some more robust results than in most previous analyses.

In short, the present paper contributes to the cliometric controversial debate on fiscal sustainability and fiscal or monetary dominance, in three important dimensions.

First, we use a very long time span to disentangle between the interaction between fiscal and monetary authorities in an attempt to demonstrate how institutional changes can have important effects on the relationship between the budget surplus-to-GDP ratio and the public debt-to-GDP ratio: the Ricardian or "monetary dominant" regime and the Non-Ricardian or "fiscal dominant" regime. This approach might be classified in the subset of studies that look for structural breaks in that break dates and regimes are determined by the data. In doing so, we use historical time series statistics for Italy during a 160-years span in which different debt crises episodes and institutional changes, ran in parallel with fiscal adjustment episodes and the use of the inflation tax.

Second, we use the methodology developed in Phillips, Wu and Yu ( Phillips et al. (2011), PWY henceforth) and ( Phillips et al. (2015a, 2015b), PSY henceforth) to examine whether the Italian public debt-GDP ratio shows a speculative bubble behavior at any point time from 1861 to 2020. This methodology implies the sustainable dynamics of public debt is interrupted by recurrent episodes of explosive public debt dynamics. That is, it represents the maintained hypothesis of the empirical analysis in order to obtain evidence in favour of a sustainable public debt process in terms of a “global” nonstationary sequence eventually interrupted by, at least, one collapsing mildly explosive episode.

Lastly, we provide a test for sustainability of the Italian government deficit and the fiscal dominance or monetary dominance over the period 1861–2020, using the FTPL. In this case, in order to control for structural breaks, we make use of recent developments in cointegrated regression models with multiple structural changes. Specifically, we use the approach proposed by Kejriwal and Perron (2008, 2010) to test for multiple structural changes in cointegrated regression models. These authors develop a sequential procedure that not only enables the detection of parameter instability in cointegrated regression models but also allows for consistency in the number of breaks present. Furthermore, we test the cointegrating relationship when multiple regime shifts are identified endogenously. In particular, the nature of the long-run relationship between the budget surplus-to-GDP ratio and the public debt-to-GDP ratio is analyzed using the residual based test for the null hypothesis of cointegration with multiple breaks proposed in Arai and Kurozumi (2007) and Kejriwal (2008).

The scheme of the paper is as follows. The literature is selectively surveyed in Sect. 2. Section 3 describes the standard empirical analysis of the long-run sustainability. The underlying theoretical framework of the fiscal theory of the price level (FTPL) is briefly described in Sect. 4. The empirical results are presented in Sect. 5. Section 6 draws the main conclusions.

## Literature review

On the one hand, our study links to previous empirical works that have assessed fiscal sustainability for the Italian economy. First, using the cointegration approach over the 1950–2002 period, Galli and Padovano (2008) find that Italian public finances fail to be compatible with sustainability requirements. Second, Piergallini and Postigliola (2012) examine the historical dynamics of government debt, from 1861 to 2009. Controlling debt dynamics for fiscal feedback policies of the Barro-Bohn approach, the debt-GDP ratio is found to be mean-reverting, perhaps due to a positive response of primary surpluses to variations in outstanding debt. More recently, Piergallini and Postigliola (2020), using a nonlinear perspective over 1861 to 2016 period, show that Italy’s primary-surplus policies are consistent with debt sustainability. Specifically, they found significant evidence in favor of the hypothesis of nonlinearity in the primary surplus-debt reaction policy function. Thus, based upon the smooth transition regression approach, they show that there exists a threshold level in the debt-GDP ratio, approximately equal to 105 percent, above which Italian fiscal policy makers are concerned with corrective actions to avoid insolvency. Finally, because of this after-threshold positive reaction of primary surpluses, they conclude that Italy’s budgetary policy is on a sustainable path.

On the other hand, our study is also related to previous empirical works that have assessed the fiscal or monetary dominance for Italian economy, using the FTPL. This approach takes into account monetary and fiscal policy interactions and assumes that fiscal policy may determine the price level, even if monetary authorities pursue an inflation targeting strategy.

The FTPL builds on the contributions of, among others, Leeper (1991); Sims (1994, 2016); Woodford (1995, 2001), and Cochrane (2001, 2021); some critical appraisals of the theory can be found, e.g., in McCallum and Nelson (2005).

The empirical evidence regarding the FTPL is abundant concerning the Euro Area, or some member countries [ Bajo-Rubio et al. (2009, 2014); Afonso and Jalles (2017), and Panjer et al. (2020), amongothers].

For the Italian case, several studies have dealt with the issue of fiscal dominance in Italian economy using a short or a long sample, but no clear evidence on the prevalence of an MD or FD regime was found. However, this type of evidence is not definitive for it is not robust to the time span or to the estimation methodology.

On the one hand, empirical studies on short sample have tended to reject of FD regime. First, Gaiotti and Salvemini (1993) using simulations of the monthly model of the Bank of Italy for the period 1980–1990, show that until 1989, a shock to the budget deficit to have only a mild and short-lived effect on money and monetary base, and after 1989, the effect is null. They conclude that there is no FD regime in 1980s–1990s. Secondly, Tullio and Ronci (1997) estimate a reaction function for the period 1970–1992 and find that the effect of the budget deficit on money growth drops in 1977 and conclude there is no evidence of FD regime after this date. Third, Gaiotti et al. (1998) estimate a structural VAR using data for the period 1985–1996. They show that in the 1990s, changes in expectations on the sustainability of public debt are not found to have had an effect on inflation and monetary policy shocks affected inflation expectations and conclude that in the 1990s there is no FD regime, i.e, this period could be a MD regime. Lastly, Gressani et al. (1988), using data for the period 1980–1986 and with simulations of the quarterly model of the Bank of Italy find that there is no FD regime, i.e, this period could be a MD regime.

On the other hand, empirical studies on long sample show the evidence of FD or MD dominance is mixed. First, Tattara and Volpe (1997) test and reject the FD regime for the period 1862–1913. They use a reduced-form equation of a model where neither public expenditures, nor taxes, nor public deficits have any role to play. Secondly, Gallo and Otranto (1998), using data for the period 1863–1994 and a Markov switching approach, find a structural break in the relationship between deficits and money around the mid-1970s. This evidence supports the hypothesis of FD regime ends since this mid-1970s. Third, Favero and Spinelli (1999), using data for the period 1875–1994 and a structural approach, find that FD regime had began to break down since 1975, and started a long period of monetary dominance regime (MD). Lastly, Fratianni and Spinelli (2001), for the period 1865–1998 and using a VAR methodology, find that the budget deficit leads the creation of monetary base by the Treasury, and conclude that FD regime has been the prevailing regime in Italy since political unification in 1861.

## The standard empirical analysis of the long-run fiscal sustainability

The sustainability of public finances, also referred to as fiscal sustainability, is the ability of government to sustain its current spending, tax and other policies in the long-run without threatening the government solvency or without defaulting on some of the government’s liabilities. In other words, fiscal sustainability requires a government to be solvent, i.e., it has to be able to repay its debt at some point in the future.

In order to describe the possible ways of achieving fiscal sustainability, we will make use of the budget identity that links the public deficit to public revenues, public spending, and stock of public debt. The public deficit is the difference between public spending and public revenues. It also equals the change in public debt. In algebraic terms, let $DEF_{t}$ denotes the total public deficit (i.e., including interest payment) in the year *t*, $T_{t}$ total revenues, $G_{t}$ the primary expenditures (i.e., excluding interest payment), $B_{t-1}$ the stock of public debt at the end of year $t-1$ (all variables in nominal terms), and $i_{t\text { }}$the long-run interest rate. The budget identities are then,

1 $${\text{DEF}}_{t} = G_{t} - T_{t} + i_{t} B_{{t - 1}}$$

2 $$B_{t}=\, B_{t-1}+DEF_{t}$$

From Eqs. (1) and (2), the nominal budget equation can be written as,

3 $$\begin{aligned} B_{t}=G_{t}-T_{t}+(1+i_{t})B_{t-1}=DEF_{t}^{0}+(1+i_{t})B_{t-1} \end{aligned}$$

where the primary public deficit $DEF_{t}^{0}$ $=G_{t}-T_{t}$.

The corresponding GDP-ratio version is,

4 $$\begin{aligned} \frac{B_{t}}{Y_{t}}=\frac{DEF_{t}^{0}}{Y_{t}}+\frac{(1+i_{t})}{(1+\gamma _{t})}\frac{B_{t-1}}{Y_{t-1}} \end{aligned}$$

where $Y_{t}$ is the nominal GDP, and $\gamma _{t}$ $=Y_{t}$/$Y_{t-1}-1$ is the nominal GDP growth rate.

Let $b_{t}$ denote a generic, scaled version of public debt (e.g., the GDP-ratio, $B_{t}$/$Y_{t}$), let $s_{t}$ denote the corresponding GDP-ratio version of the primary public surplus ($-DEF_{t}^{0}/Y_{t}$), and let $r_{t}$ denote the corresponding GDP-ratio version of the "return" on public debt, e.g., $r_{t}=(1+i_{t})/(1+\gamma _{t})-1\approx i_{t}-\gamma _{t}$.

The dynamic of public debt can be described compactly as,

5 $$\begin{aligned} b_{t}=(1+r_{t})b_{t-1}-s_{t} \end{aligned}$$

From Eq. (5), we can readily compute the paths of public debt implied by arbitrary sequences of public primary surplus and interest payments. Iterating backward gives the following expression which mainly serves as the starting point for the theoretical analysis, with relevant empirical implications for fiscal sustainability, in Bohn (1998, 2008),

6 $$\begin{aligned} b_{t}^{*}=\sum \limits _{j=0}^{\infty }\frac{1}{(1+r)^{j}}E_{t}\left[ s_{t+j}\right] +\lim _{n\longrightarrow \infty }\frac{1}{(1+r)^{n}}E_{t}\left[ b_{t+n}\right] \end{aligned}$$

where $b_{t}^{*}=(1+r_{t})b_{t-1}$ denotes public debt at the start of period *t* and where $E_{t}$ denotes conditional expectations.

Equation (6), shows that initial public debt equals the expected present value of future primary public surpluses if and only if discounted future public debt converges to zero. That is,

7 $$\begin{aligned} b_{t}^{*}=\sum \limits _{j=0}^{\infty }\frac{1}{(1+r)^{j}}E_{t}\left[ s_{t+j}\right] \end{aligned}$$

and is equivalent to the current value of future public debt being convergent to 0,

8 $$\begin{aligned} \lim _{n\longrightarrow \infty }\frac{1}{(1+r)^{n}}E_{t}\left[ b_{t+n}\right] =0 \end{aligned}$$

Equation (7) is commonly known as the Intertemporal Budget Constraint (IBC) and Eq. (8) as the Transversality Condition (TC) of the intertemporal decision problem of the government.

On the one hand, according to Eq. (7), the condition for fiscal sustainability requires that the government must run expected future budget surpluses equal, in present-value terms, to the current value of its outstanding debt. On the other hand, the TC (8), rules out a Ponzi scheme (whereby debt is perpetually rolled over) as the necessary condition for lenders to hold government bonds. Therefore, it implies a sustainable trajectory of public debt and an explosive behavior only temporary, i.e., absence of bubbles.

The usual procedure in most of the empirical contributions on the long-run sustainability of budget deficits consists of testing the government’s IBC. The results, however, are sometimes inconclusive due to differences in the econometric methodology, the particular specification of the TC, and the sample period used. A common criticism to most of the available literature is that the econometric procedures used require a large number of observations, which is not usually the case in most tests of the IBC. There is a large literature on the topic, though empirical tests of solvency (or fiscal sustainability), have gone through different stages.

## The fiscal theory of the price level

### The interactions between monetary and fiscal policies

The traditional macroeconomic approach assumes that the fiscal authority sets primary surpluses in order to assure fiscal solvency, for a given price level. In this way, the monetary authority is expected to set the price level, without facing any constraint. This scenario is referred in the literature as the Ricardian or “ monetary dominant” (MD) regime, and works as follows: monetary policy would be “ active”, being price determination its nominal anchor; whereas fiscal policy would adjust according to a Ricardian rule in a “ passive” way, so that the budget surplus path would be endogenous.

The emergence in the 1990s of the fiscal theory of the price level (hereafter, FTPL) has however challenged this view. The FTPL indeed provides a theoretical determination of the price level with strong emphasis on the links between monetary and fiscal policies (Leeper 1991), in both purely flexible and sticky prices frameworks (Woodford 1995, 1998) and without resorting to seigniorage or monetization arguments. The FTPL links price determination to the government present value budget constraint, i.e. the equality of the public debt with the present discounted value of future expected primary surpluses. The key intuition of the FTPL is that, if current and future fiscal policies are set without concern for sustainability, the general price level will “ jump” in order to fulfill the present value budget constraint (Woodford 2001). This is the so-called non-Ricardian or “ fiscal dominant” (FD) regime. In such a context, the budget surplus would be exogenous, and the endogenous adjustment of the price level would be required in order to guarantee fiscal solvency in terms to satisfied Eq. (7).

On the one hand, according to the traditional macroeconomic approach, the price level would be determined in the money market, following the quantity theory of money, and the primary surplus would adjust endogenously to satisfy the present-value budget constraint. In terms of Eq. (7) and Eq. (8), $s_{t}$ would be set to meet a given $b_{t}$, independently of the price level. The interdependence between monetary and fiscal policy can still appear as follows (see Sargent and Wallace (1981). Assume that, in Eq. (7), seigniorage is allowed, so that $b_{t}$ would denote all the government’s liabilities, and $s_{t}$ include the seigniorage revenue. Hence, if $b_{t}$ is given and the government wants to reduce the primary surplus, seigniorage must be increased keeping the total $b_{t}$ constant, leading to a higher inflation. In this way, the requirements of fiscal solvency can mean a limit to the options open to the central bank. The corollary of this argument would be the now standard recommendation of granting independence to the central bank, which should assign a high priority to inflation, and strictly commit to understandable and publicized rules when conducting monetary policy. As a consequence, seigniorage eventually has disappeared as a source of budget deficit financing in advanced countries.

On the other hand, according to the conventional approaches consider that the price level may be predominantly determined by budgetary decisions relating to public debt and future primary balances (Leeper 1991; Sims 1994; Woodford 1995; Cochrane 2021). Following this theoretical approach, an increase in inflation results from budget expansions that do not consider future counterparts, such as increasing taxes or decreasing expenditure, thus implying that the increase in debt will be paid through inflation (Woodford 1995; Sims 2016). In light of this theory, monetary policy may be insufficient or secondary in determining the equilibrium price level.

If inflation expectations are anchored to fiscal policy decisions (Woodford 1995), expansionary monetary policy measures may prove ineffective if there is no anticipation of tax cuts or fiscal expansions (Sims 2016). According to Sims (2016), low or negative interest rates will not have inflationary effects if budget options point to debt reduction.

The traditional macroeconomic analysis assumes that monetary policy should play a central role in stabilizing the price level. However, inflation will also depend on the interaction between monetary policy and fiscal policy: monetary policy measures have budgetary implications, and fiscal policies can influence real economic activity and public debt dynamics. That is, an expansionary fiscal policy could boost economic activity, with a positive impact on inflation via the Phillips curve, or lead to an explosive dynamic of public debt that implies financing of public deficit via monetization.

Considering the recent context of the eurozone, and taking into account the austerity measures adopted in some countries as a response to the sovereign debt crisis and, more recently, to the COVID-19 crisis and the new shock generated by Russia-Ukraine war. As well as the fact that, the desynchronization between monetary policy and fiscal policy may have mitigated the potential effects of the conventional and unconventional accommodative measures adopted by the ECB. If monetary policy proves to be insufficient in meeting the target inflation, this may suggest the need for a more active – or central – role for fiscal policy, as assumes the FTPL.

### The basic model of the FTPL

Following to Cochrane (2021), we can write the Eq. (7) in real terms as,

9 $$\begin{aligned} \frac{B_{t-1}}{P_{t}}=E_{t}\sum \limits _{j=0}^{\infty }\beta ^{j}s_{t+j} \end{aligned}$$

where the left side of the equation is the real value of public debt, defined by its nominal value with a maturity of one period, $B_{t-1}$, and the price level, $P_{t}$, which will have to be equal to the present value of the real primary surpluses futures, $s_{t+j}$, with subjective discount factor $\beta ^{j}$ or real interest rate *R*. The basic fiscal theory Eq. (9) quickly generalizes to say that the real value of nominal debt equals an infinite present value of surpluses. The price level adjusts so that the real value of nominal debt equals the present value of future surpluses. i.e given *B* and *s*, *P* would "jump" to satisfy (9). In other words, if the market believes the government’s commitment when setting *s*, a value of *P* will be set so that *B* was not excessive and (9) could be satisfied.

Assuming an active role of fiscal policy in determining the price level, the FTPL implicitly suggests a non-Ricardian regime: the sequence of primary surpluses is determined without considering the need to ensure fiscal solvency (Woodford 1995). If fiscal policy is "irresponsible", and implies an explosive debt dynamic, the stabilization of public debt will only be ensured with an adjustment in the price level. Under this non-Ricardian hypothesis, monetary policy is not sufficient to ensure target inflation rate and the equilibrium price level will be determined by budgetary policies (Woodford 1995).

It is important to emphasize that, from the perspective of the FTPL, public deficits and high levels of public debt do not necessarily translate into increases in the price level (Cochrane 2021). Ceteris paribus, the price level will be determined by the relationship between the current level of public debt and the expected present value of all future public surpluses.

To test empirically the prevalence of monetary dominance versus fiscal dominance in the basic FTPL Eq. (9), the literature has traditionally resorted to the backward-looking approach proposed by Bohn (1998), which would imply that, in a Ricardian or MD regime, an increase in the previous level of debt would result in a larger primary surplus today. This approach also provides an indirect test on the solvency of public finances.

According to the Bohn’s approach, we aim to estimate the linear cointegrating relationship between the primary public surplus and the (lagged) level of public debt via the following fiscal reaction function,

10 $$\begin{aligned} s_{t}=\alpha +\gamma b_{t-1}+\varepsilon _{t} \end{aligned}$$

where $\varepsilon _{t}$ is an error term that satisfies standard assumptions of zero mean and constant variance. In this equation, a positive estimate of $\gamma$ would be a sufficient condition for solvency, indicating that the government satisfies its present-value budget constraint; that is, in terms of the TC, $s_{t}$ would be set to meet a given $b_{t}$, independently of the price level, $P_{t}$. Furthermore, an estimated $\gamma >0$ would indicate the prevalence of an MD regime, and an estimated $\gamma \le 0$ the prevalence of an FD regime (or FTPL regime).

## Empirical results

In this section, we will provide a formal test of the sustainability of the Italian government deficit over the period 1861–2020; and, more importantly, we will analyze the role played by monetary and fiscal dominance in order to get fiscal solvency along the period.

### Data

We consider a long historical time series in which many fiscal crisis events are known to have occurred. The length of this database makes it particularly suitable for the econometric approach adopted in this paper (160 years).

The data and sources are:

- 1861–1994: a) the public debt-to-GDP ratio, $b_{t}$, from Piergallini and Postigliola (2012, 2020); the primary (i.e., excluding interest payments) budget surplus-to-GDP ratio, $s_{t}$, from Piergallini and Postigliola (2012, 2020).
- 1995–2020; b) the public debt-to-GDP ratio, $b_{t}$, Commission (2021); the primary (i.e., excluding interest payments) budget surplus-to-GDP ratio, $s_{t}$, from Commission (2021).^1^

**Fig. 1:**
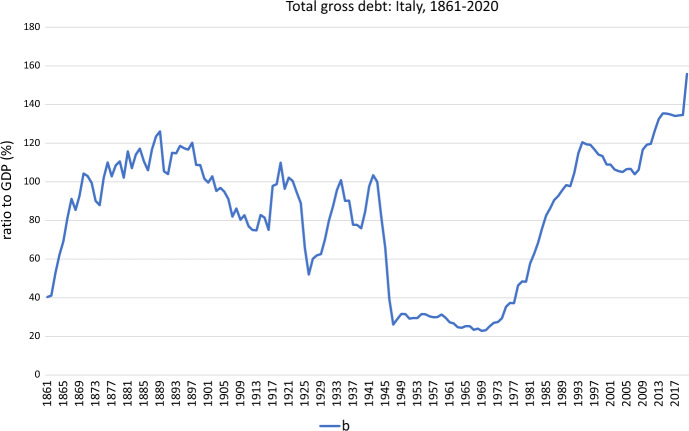
Total gross debt: Italy, 1861–2020

**Fig. 2:**
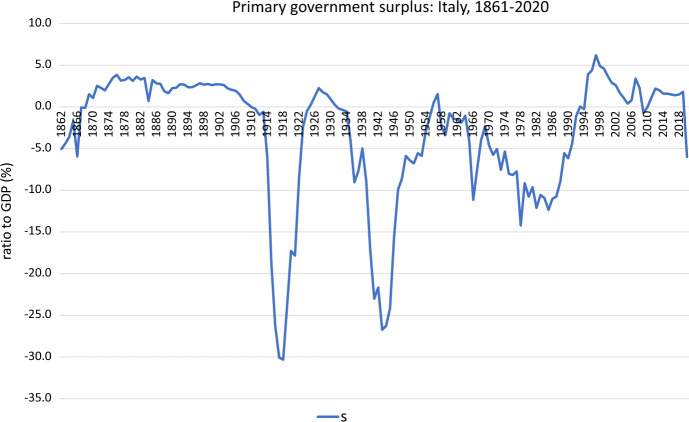
Primary government surplus: Italy, 1861–2020

**Fig. 3:**
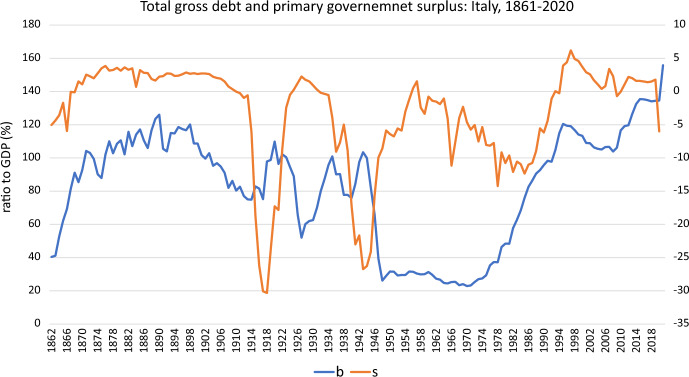
Total gross debt and primary governemnet surplus: Italy, 1861–2020

In our empirical analysis, we use annual data of the Italian economy for the period 1861–2020 (post-unification Italy). Given that the present analysis is going to focus on the Italian case, we think that it is necessary to sketch the brief historical budgetary background. We can broadly follow dynamics of the path the Italian public debt, as % of GDP, and the primary budget surplus, as % of GDP, between 1861 and 2020 in Figs. 1 and 2, respectively, where the expansions of public debt and public deficit peaks are markedly visible in them. The time evolution of both series appears in Fig. 3, showing a close co-movement between the two series. However, the plot also suggests that the association between $b_{t}$ and $s_{t}$ may have altered over time.

In general, with the exception of the first three years of the new Kingdom in the aftermath of the political unification (from 1861 to 1863), of one year in the Fascist period (1926), and of the first thirty-six years in the post-World War II period (from 1946 to 1981), the dynamics of the Italian public debt-to-GDP ratio, $b_{t}$, has stayed significantly above the value of 60 percent, the threshold ratio imposed by the Maastricht Treaty in the euro area, as showed in Fig. 1. The COVID-19 crisis has pushed the Italian public debt to a recent all-time high of 155.8 percent of GDP in 2020.

On the other hand, high negative peaks of the Italian primary government deficits are reached by the world war-time periods, and in 2020 as consequence of the COVID-19 crisis. Conversely, some periods of positive budget deficits are found: from 1869 to 1876 during the "Historical Right" period, from 1922 to 1927 during the Fascist period, from 1952 to 1957, during the "Golden Age", from 1995 to 2001, during the Maastricht period, from 2002 to 2004, during the second Berlusconi government, from 2006 to 2008, during the last Prodi government, from 2011 to 2013, during the Monti government; and from 2014 to 2019, during different governments.

A more detailed account of the evolution of the Italian public finances over this period can be found in De Cecco (1996); De Cecco and Pedone (1996); Piergallini and Postigliola (2018); Postigliola and Strangio (2017), and Piergallini and Postigliola (2020).

In addition, when looking into how these deficits were financed, the key role (whether directly or indirectly) played by the Bank of Italy could lead us to presume that monetary policy had been subordinated to the evolution of fiscal policy during most of the period of analysis. Firstly, from 1861 to the 1980s, monetary policy was to be dominated by the stance of fiscal policy, i.e, was subordinate to the needs of financing the budget deficit. Secondly, this fiscal dominance of monetary policy was only broken in the early 1980s, when the Bank of Italy gradually acquired greater independence in setting monetary policy, and did so independently of fiscal decisions. The process was completed in the 1990s, when in 1992 the Bank of Italy was granted full instrument independence by allowing it to set the discount rate; and since then the objective has been to achieve inflation convergence with the Euro Area.

A more detailed for a summary and discussion of this interdependence between monetary and budgetary policy in Italian economy over this period can be found in Muscatelli and Spinelli (2000).

### Order of integration analysis and structural breaks of the time series

Given previous analyses in the literature and the expected effects of the different economic crises that might have affected the variables that we are dealing with, we start the analysis of the order of integration of the time series involved in our study investigating the presence of structural breaks. This is an important feature. Unit root tests can lead to misleading conclusions if the presence of structural breaks is not accounted for when testing the order of integration. Trend breaks appear to be prevalent in macroeconomic time series, and unit root tests, therefore need to make allowances for these breaks if they are to avoid the serious effects that unmodelled trend breaks have on power. In a seminal paper, Perron (1989) shows that failure to account for trend breaks present in the data results in unit root tests with zero power, even asymptotically. Consequently, when testing for a unit root, allowing for this kind of deterministic structural change has to become a matter of regular practice. To avoid this pitfall, we run tests to assess whether structural breaks are present or not in $b_{t}$ and $s_{t}$ series over the full sample.

In our paper, we have used the GLS-based unit root tests with multiple structural breaks under both the null and the alternative hypotheses proposed in Carrion-i-Silvestre et al. (2009). The commonly used tests for unit root with a structural change in the case of an unknown break date (Perron 1997), assume that if a break occurs, it does so only under the alternative hypothesis of stationarity. The methodology developed by Carri ón-i-Silvestre et al. (2009) solves many of the topical problems in standard unit root tests with a structural change in the case of an unknown break date. Carrion-i-Silvestre et al. (2009) consider the modified unit root tests (*M*-class tests) analysed by Ng and Perron (2001).

Carrion-i-Silvestre et al. (2009) consider three models: Model 0 (“ level shift” or “ crash”), Model I (“ slope change” or “ changing growth”), and Model II (“ mixed change”). In our empirical application we have used the Model 0 and the Model II for $s_{t}$ and $b_{t}$ series, respectively.

The results of applying the Carrion-i-Silvestre-Kim-Perron tests to Model 0 are shown in Table 1, allowing for up to three breaks. As Table 1 shows, the null hypothesis of a unit root with three structural breaks that affects the level (intercept) or the level and slope of the times series (mixed change) cannot be rejected by any of the tests at the 1% level of significance.^2^ Consequently, we can conclude that the $s_{t}$ and $b_{t}$ variables are *I*(1) with three different structural breaks.

**Table 1:** *M* unit root tests with multiple structural breaks of Carrion-i-Silvestre et al. (2009)^a,b,c^

Variable	Model	$MZ_{\alpha }^{GLS}$	$MZ_{t}^{GLS}$	$MSB^{GLS}$	$MP_{T}^{GLS}$
$s_{t}$	0	−18.15	−3.01	0.165	15.70
$b_{t}$	II	−26.22	−3.57	0.136	11.27

^a^Unit root tests with multiple structural breaks under both the null and the alternative hypotheses. A ** denote significance at the 5% level

^b^The structural break affects the level (Model 0). The structural break affects the level and the slope of the time trend (Model II)

^c^The critical values were obtained by simulations using 1000 steps to approximate the Wiener process and 10,000 replications

### Structural changes in the variance of the time series

The second step in our analysis is to examine the time series properties of the series by testing structural changes in the variance over the full sample. These testing problems are important for practical applications in macroeconomics and finance (including fiscal variables) for detecting structural changes in the variability of shocks in time series. In empirical applications based on linear regression models, structural changes often occur in both the error variance and regression coefficients, possibly at different dates. From an applied perspective the existence of breaks in variance has also attracted considerable interest following the work of McConnell and Perez-Quiros (2000) who documented the existence of a break in U.S. output volatility occurring in the early mid 1980s. Building on this line of research, Sensier and van Dijk (2004) also explored the existence of a break in the volatility of a large database of U.S. macroeconomic series and found that the vast majority of the real series were also characterized by a variance shift that occurred during the early mid 1980s; see also Perron and Yamamoto (2022), and Stock and Watson (2002).

We have used the test statistics to test jointly for structural changes in mean and variance proposed by Perron et al. (2020). More specifically, these authors provided a comprehensive treatment of the problem of testing jointly for structural changes in both the regression coefficients and the variance of the errors in a single equation regression model involving stationary regressors, allowing the break dates for the two components to be different or overlap.

Perron et al. (2020) consider several types of test statistics for testing structural changes in mean and/or variance: (1) the $\sup LR_{T}$ test statistic for *m* coefficient changes given no variance changes; (2) the $\sup LR_{1,T}$ test statistic for *n* variance changes given no coefficient changes; (3) the $\sup LR_{2,T}$ test statistic for *n* variance changes given *m* coefficient changes; (4) the $\sup LR_{3,T}$ test statistic for *m* coefficient changes given *n* variance changes; (5) the $\sup LR_{4,T}$ test statistic for *m* coefficient changes and *n* variance changes; (6) The $UD\max$ tests for each version can be computed by taking a maximum over a range of $1\le n\le N$ for $\sup LR_{1,T}$ and $\sup LR_{2,T}$, over a range of $1\le n\le M$ for $\sup LR_{T}$ and $\sup LR_{3,T}$, and over ranges of $1\le n\le N$ and $1\le m\le N$ for the $\sup LR_{4,T}$; (7) the $\text {seq}LR_{9,T}$ test statistic for *m* coefficient changes versus $m+1$ coefficient changes given *n* variance changes; (8) the $\text {seq}LR_{10,T}$ test statistic for *n* variance changes versus $n+1$ variance changes given *m* coefficient changes. *M* and *N* denotes the maximum number of breaks for the coefficients and the variance, respectively.

First, we investigate structural changes in the conditional mean and in the error variance of $b_{t}$ (see Fig. 1). We use $M=3$ and $N=2$ and take into account any potential serial correlations in the error term via a HAC variance estimator following Bai and Perron (1998). Table 2a reports the $\sup LR_{4,T}$ and the $UD\max$ $LR_{4,T}$ tests. The results do not suggest rejections of the null hypothesis of no breaks jointly in the conditional mean and in the error variance. Table 2b presents the results when testing for changes in the coefficients, allowing for changes in the variance. Using $\sup LR_{3,T}$ and $UD\max$ $LR_{3,T}$ tests, we obtain no evidence of a change in the conditional mean coefficients. The sequential procedure using the $\sup LR_{9,T}$ test confirms no structural changes in mean. Table 2c presents the results of the $\sup LR_{2,T}$ and the $UD\max$ $LR_{2,T}$. These suggest the presence of breaks in the variance. The sequential test $\sup LR_{10,T}$ suggests 2 breaks at 1945 and 1978 when $m_{a}=0$ and $m_{a}$ $=1$. Hence, for the public debt-to-GDP ratio, $b_{t}$, we conclude for 2 structural changes in the error variance and no change in the conditional mean. The changes are such that the variance fall considerably in the period 1946–1978 but has risen very significantly again in the period 1979–2020.

**Table 2:** Tests for structural changes in mean and variance from Perron et al. (2020)^d,e^: Italian public debt, $b_{t}$

(a) Tests for structural changes in mean and/or variance^a^							
	$\sup {LR}_{4,T}$			${UD}\max {LR}_{4,T}$			
	${m}_{a}{=1}$	${m}_{a}{=2}$	${m}_{a} {=3}$	${M=3,N=2}$			
${n}_{a}{=1}$	1.17	2.18	0.42	4.56			
${n}_{a}{=2}$	2.12	1.46	4.56				

(b) Tests for structural changes in mean^b^							
	$\sup {LR}_{3,T}$			${UD}\max {LR}_{3,T}$	$\text {seq}{LR}_{9,T}$		
	${m}_{a}{=1}$	${m}_{a}{=2}$	${m}_{a} {=3}$	${M=3}$	${m}_{a}{=1}$	${m}_{a}{=2}$	${m}_{a}{=3}$
${n}_{a}{=0}$	2.63	3.60	2.88	3.60	3.12	2.53	2.49
${n}_{a}{=1}$	0.01	3.66	2.16	3.66	4.06	15.15$^{2}$	2.49
${n}_{a}{=2}$	0.05	0.78	0.77	0.78	4.08	2.53	4.94

(c) Tests for structural changes in variance^c^							
	$\sup {LR}_{2,T}$		${UD}\max {LR} _{2,T}$	$\text {seq}{LR}_{10,T}$			
	${n}_{a}{=1}$	${n}_{a}{=2}$	${N=2}$	${n}_{a}{=1}$	${n}_{a}{=2}$	Break dates	
${m}_{a}{=0}$	9.76$^{2}$	5.80	9.76$^{2}$	6.39	7.11	1945	
${m}_{a}{=1}$	12.95$^{2}$	8.23$^{2}$	12.95$^{2}$	218.5$^{3}$	12.21$^{2}$	1978	
${m}_{a}{=2}$	−0.59	4.00	4.00	16.07$^{3}$	15.67$^{3}$		
${m}_{a}{=3}$	−1.12	3.55	3.55	3.06	15.67$^{3}$		

^a^Testing for structural changes in either or both the regression coefficients (mean) and the error variance. Hypothesis null of no breaks

^b^Testing for structural changes in the regression coefficients (mean), allowing for changes in the error variance. Hypothesis null of no breaks

^c^Testing for structural changes in the error variance, allowing for changes in the regression coefficients (mean). Hypothesis null of no breaks.

^d^Superscripts $^{1,2,3}$ indicate significance at the 10%, 5% and 1% levels, respectively

^e^The critical values are taken from Bai and Perron (1998); Perron et al. (2020), and Perron and Yamamoto (2022)

Second, we investigate structural changes in the conditional mean and in the error variance of $s_{t}$ (see Fig. 2). We also use $M=3$ and $N=2$ and take into account any potential serial correlations in the error term via a HAC variance estimator. Table  3a presents the results for the $\sup LR_{4,T}$ and the $UD\max$ $LR_{4,T}$ tests. The results do not suggest rejections of the null hypothesis of no breaks jointly in the conditional mean and in the error variance. Table 3b presents the results when testing for changes in the coefficients, allowing for changes in the variance. We obtain strong evidence of no change in the conditional mean coefficients. The sequential procedure, using the $\sup LR_{9,T}$ test, confirms these results. Table 3c presents the results of the $\sup LR_{2,T}$, the $UD\max$ $LR_{2,T}$, and the sequential test $\sup LR_{10,T}$ tests. These results suggest the presence of breaks in the variance with a single break date estimated in 1913. Hence, for the primary budget surplus-to-GDP ratio, $s_{t}$, we obtain a structural change in the error variance and no change in the conditional mean. In this case, the variance has risen considerably in the period 1914–2020.

**Table 3:** Tests for structural changes in mean and variance from Perron et al. (2020)^d,e^: Italian primary government surplus, $s_{t}$

(a) Tests for structural changes in mean and/or variance^a^							
	$\sup {LR}_{4,T}$			${UD}\max {LR}_{4,T}$			
	${m}_{a}{=1}$	${m}_{a}{=2}$	${m}_{a} {=3}$	${M=3,N=2}$			
${n}_{a}{=1}$	0.16	2.76	1.65	2.76			
${n}_{a}{=2}$	1.30	2.54	2.09				

(b) Tests for structural changes in mean^b^							
	$\sup {LR}_{3,T}$			${UD}\max {LR}_{3,T}$	$\text {seq}{LR}_{9,T}$		
	${m}_{a}{=1}$	${m}_{a}{=2}$	${m}_{a} {=3}$	${M=3}$	${m}_{a}{=1 }$	${m}_{a}{=2}$	${m}_{a}{=3}$
${n}_{a}{=0}$	3.03	3.62	2.76	3.62	5.16	5.33	6.10
${n}_{a}{=1}$	1.29	1.88	1.13	1.88	5.16	5.33	6.10
${n}_{a}{=2}$	0.01	1.52	0.91	1.52	0.87	6.10	6.10

(c) Tests for structural changes in variance^c^							
	$\sup {LR}_{2,T}$		${UD}\max {LR} _{2,T}$	$\text {seq}{LR}_{10,T}$			
	${n}_{a}{=1}$	${n}_{a}{=2}$	${N=2}$	${n}_{a}{=1}$	${n }_{a}{=2}$	Break dates	
${m}_{a}{=0}$	3.81	4.98	4.98	6.44	9.04		
${m}_{a}{=1}$	7.85$^{1}$	4.85	7.85	6.44	9.04	1913	
${m}_{a}{=2}$	5.30	6.49	6.49	10.66$^{1}$	10.00$^{1}$		
${m}_{a}{=3}$	4.04	7.06$^{1}$	7.06	15.44$^{3}$	10.01$^{1}$		

^a^Testing for structural changes in either or both the regression coefficients (mean) and the error variance. Hypothesis null of no breaks

^b^Testing for structural changes in the regression coefficients (mean), allowing for changes in the error variance. Hypothesis null of no breaks

^c^Testing for structural changes in the error variance, allowing for changes in the regression coefficients (mean). Hypothesis null of no breaks

^d^Superscripts $^{1,2,3}$ indicate significance at the 10%, 5% and 1% levels, respectively

^e^The critical values are taken from Bai and Perron (1998); Perron et al. (2020), and Perron and Yamamoto (2022)

The volatile behavior of fiscal policy and the associated loss of credibility have often been responsible for their recurrent crises. There is body of evidence that fiscal policy is not conducted by benevolent governments trying to maximize a social welfare function. Fiscal policy is too volatile and in some countries there are acyclical or even procyclical policies. Furthermore, the lack of internalization of spending decisions leads to growing fiscal deficits and accumulation of public debt. This behavior leads to excessive macroeconomic volatility, and in turn, this volatility affects the long-term growth of the country. Finally, restrictions on fiscal policy (explicit or implicit, fiscal rules) reduce this volatility and provide verifiable macroeconomic benefits; see, e.g., Fatás and Mihov (2008) and the references therein.

### The potential explosive public debt dynamics

In order to detect episodes of potential explosive behavior in the Italian public debt, $b_{t}$, we use recent recursive unit root tests for explosiveness proposed by PWY and PSY. They developed a new recursive econometric methodology for real-time bubble detection that proved to have a good power against mildly explosive alternatives.

The sustainable dynamics of public debt implies that $b_{t}$ is a process integrated *I*(1) that is interrupted by recurrent episodes of explosive public debt dynamics. That is, it represents the maintained hypothesis of the empirical analysis in order to obtain empirical evidence in favour of a sustainable public debt process in terms of a “ global” nonstationary sequence eventually interrupted by, at least, one collapsing mildly explosive episode.

First, PWY proposed a $\sup ADF$ (*SADF*) statistic to test for the presence of explosive behavior in a full sample. Second, PSY developed a double-recursive algorithm that enable bubble detection and consistent estimation of the origination (and termination) dates of bubble expansion and crisis episodes while allowing for the presence of multiple structural breaks within the sample period. They showed that when the sample includes multiple episodes of exuberance and collapse, the PWY procedures may suffer from reduced power and can be inconsistent, thereby failing to reveal the existence of bubbles. This weakness is a particular drawback in analyzing long time series or rapidly changing of data where more than one episode of explosive behavior is suspected. PSY also proposed a generalized version of the $\sup ADF$ (*SADF*) test of PWY, based on the $\sup$ value of the *BSADF*. This statistics is referred to as *GSADF* and is used to test the null of a unit root against the alternative of recurrent explosive behavior.

For our empirical application, the lag order *K* is selected by using the Bayesian information criterion (BIC) with a maximum lag order of 5. We set the smallest windows size according to the rule $r_{0}=0.01+1.8/\sqrt{T}$ recommended by PSY, giving the minimum length of a sub-sample as 24 years. The origination (termination) of an explosive episode is defined as the first chronological observation for which test statistic exceed (falls below) its corresponding critical value.

**Table 4:** Tests for explosive behavior in the Italian public debt^a,b,c^, $b_{t}$

Unit root tests	Estimated value	Finite critical value		
		1%	5%	10%
*GSADF*	5.017^3^	2.474	2.042	1.791

^a^Test the null of a unit root against the alternative of recurrent explosive behavior

^b^Superscripts $^{1,2,3}$ indicate significance at the 10%, 5% and 1% levels, respectively

^c^The critical values are obtained using a Monte Carlo simulation with 2,000 replications

Table 4 reports the *GSADF* test for the null hypothesis of a unit root against the alternative of an explosive root in the Italian public debt-to-GDP-ratio series. The various critical values for this test are also reported. We conduct a Monte Carlo simulation with 2000 replications to generate the *GSADF* statistic sequences and the corresponding critical values at the 10, 5 and 1 per cent levels. As seen in Table 4, we can reject the unit root null hypothesis in favour of the explosive alternative at the 1% significance level for the *GSADF* test which test exceeds its respective 10%, 5% and 1% right-tail critical values, giving evidence that Italian public debt had explosive subperiods. Consequently, we can conclude that there is some evidence of bubbles in Italian public debt over GDP ratio.

**Fig. 4:**
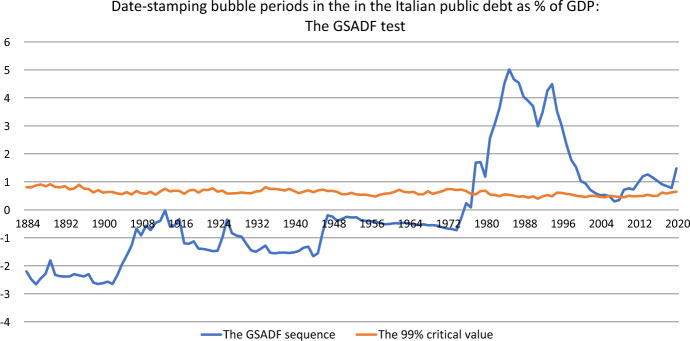
Date-stamping bubble periods in the in the Italian public debt as % of GDP: The GSADF test

Next, we conduct a real-time bubble monitoring exercise for the Italian public debt using the PSY strategy. The PSY procedure also has the capability to identify downturns and adjustments in the public debt-to-GDP ratio. To locate the origin and conclusion of the explosive behavior and the adjustments episodes, Fig. 4 plots the profile of the *GSADF* statistic for the public debt-to-GDP ratio series. We compare the *GSADF* statistic with the 99% *GSADF* critical value for each observation of interest. The initial start-up sample for the recursive regression covers the period 1831–1854 (14% of the full sample). Figure 4 identifies two episodes of explosive public debt behavior, and it permits us to date-stamp their origination and termination, as well as the potential fiscal adjustments. According to Fig. 4, there is speculative bubble behavior in the Italian public debt to-GDP ratio series over the period 1861–2020.

The first episode dated in 1977–1994. This period of explosive behavior is related to the start of high expansions in public debt over the 1980s and the early 1990s, following the so-called “divorce” between the Treasury and the Bank of Italy in 1981, removing the obligation for the central bank to buy unsold Treasury Bills at auctions. The greatest growth in the debt occurred during the 1980s and yearly 1990s, where the public debt-to-GDP ratio was gradually increased from 37.2% in 1977 to 120.5 % in 1994, even in the absence of economic recessions but with a significant increase of the yield of government bonds. The unfitness to stabilize public debt dynamics on the part of the fiscal authorities led to loss of confidence on the part of foreign investors, triggering a currency and financial crisis that caused a heavy devaluation of the Italian lira in 1992 compared to the Deutsche mark and, subsequently, the exit of Italy from the European Monetary System.

The second episode, occurred in 1995–2007, was a fiscal adjustment. The public debt was stabilized (the public debt-to-GDP ratio was gradually decreased from 120.5 % in 1994 % to 103.9% in 2007) after that an increase in real GDP growth was accompanied by a period of reduction in the yield of government bond, as a result of Italy’s accession to the Maastricht Treaty.

The third episode detected is a second period of explosive debt behavior (2008–2020). It is associated with the deep economic recession of 2008–2013 in the aftermath of the international financial crisis of 2007–2008, and the negative budgetary consequences (current and futures) of the recent international economic crisis triggered by COVID-19 pandemic in 2020. In this period the public debt-to-GDP ratio rose from 103.9% in 2007 to 155.8% in 2020. This level is the all-time high in 160 years in Italian economy, even when the primary public spending over GDP has remained substantially unchanged until 2019 and the primary surplus-GDP ratio up to a level on average + 4.3% annually in 2012–2019.

On the one hand, such increasing of the public debt-to-GDP ratio is partly due to a gradual decline in the real GDP growth rate, which reached $-$5.5 percent in 2009 in the aftermath of the financial crisis started in 2007–2008, and partly due to a sharp increase in public debt service following the contagion effects of the European sovereign debt crisis of 2009–2012 erupted in Greece and Ireland, and then expanded to Spain and Portugal. On the other hand, in 2020 the Italian primary government deficits has reached the -6% over GDP as consequence of the COVID-19 crisis (primary surplus over GDP of +1.8%% in 2019), and the Italian public debt has pushed to an all-time high of 155.8 % over GDP (134.6% in 2019).

### Cointegration relationships

In this section, we estimate a cointegration the long-run or relationship between $s_{t}$ and $b_{t}$. The parameter of interest is $\beta$ in Eq. (10).

First, we estimate and test the coefficients of the cointegration equation by means of the dynamic ordinary least squares (DOLS) method of Stock and Watson (1993) and following the methodology proposed by Shin (1994). This estimation method provides a robust correction to the possible presence of endogeneity in the explanatory variables, as well as serial correlation in the error terms of the OLS estimation. Additionally, to overcome the problem of the low power in classical cointegration tests in the presence of persistent roots in the residuals from the cointegration regression, Shin (1994) suggests a new test in which the null hypothesis is that of cointegration. Therefore, in the first place, we estimate a long-run dynamic equation that includes the leads and lags of all the explanatory variables, i.e., the so-called DOLS regression:

11 $$\begin{aligned} s_{t}=c+\Phi t+\gamma b_{t-1}+\sum \limits _{j=-q}^{q}\gamma _{j}\Delta b_{t-1-j}+\upsilon _{t} \end{aligned}$$

If there is cointegration in the demeaned specification given in (11), such cointegration would occur when $\Phi$ $=0$, which corresponds to deterministic cointegration and implies that the same cointegrating vector eliminates both the deterministic and stochastic trends. However, if the linear stationary combinations of *I*(1) variables have nonzero linear trends (which occurs when $\Phi \ne 0$), as given in (11), this would correspond to a stochastic cointegration. In both cases, the parameter $\gamma$ is the estimated long-run cointegrating coefficient between $s_{t}$ and $b_{t}$.

**Table 5:** Estimation of long-run relationships: tests for cointegration from Stock and Watson (1993) and Shin (1994)^a,b,c,d^

Parameter estimates	Model without structural breaks	Two-breaks model		
	Full sample	First regime	Second regime	Third regime
	1861–2020	1861–1909	1910–1973	1974–2020
$\alpha$	−11.67	−5.86	−0.89	−11.78
	(1.775)	(1.701)	(2.773)	(1.753)
$\gamma$	0.102	0.07	−0.107	0.128
	(0.020)	(0.016)	(0.041)	(0.015)
Test:				
$C_{\mu }$	0.109	0.162	0.046	0.029

^a^Standard errors are in parentheses. An AR(2) error was used for the calculation of the standard errors

^b^We choose $q=$ $INT\left( T^{1/3}\right)$ as proposed in Stock and Watson (1993)

^c^$C_{\mu }$ is *LM* statistics for cointegration using the DOLS residuals from deterministic cointegration, as proposed in Shin (1994). The null hypothesis of deterministic cointegration versus the alternative hypothesis of no deterministic cointegration

^d^Superscripts $^{1,2,3}$ indicate significance at the 10%, 5% and 1% levels, respectively. The critical values are taken from Shin (1994), Table 1, from $m=1$

The coefficient from the DOLS regression and the results of the Shin test are reported in Table 5. The null of deterministic cointegration between $s_{t}$ and $b_{t}$ is not rejected at the 1% level, with an estimated value for $\gamma$ of 0.102. Moreover, the estimated coefficient is positive and significantly different from zero at the 1% level. Accordingly, public finances would have been sustainable over the long-run and a Ricardian or MD regime as suggest the traditional macroeconomic approach, would have prevailed for Italian economy, at least for full sample (1861–2020). Therefore, the whole period can not be characterized as one of fiscal dominance (non-Ricardian or FD regime) as suggest the FTPL theory.

Our results are in line with other similar empirical works that suggest there is evidence in favor the existence of a Ricardian or MD regime on the Euro area or on some member countries [ Bajo-Rubio et al. (2009, 2014); Afonso and Jalles (2017), and Panjer et al. (2020), among others], and for Italy for some periods [ Gressani et al. (1988); Gaiotti and Salvemini (1993); Tattara and Volpe (1997); Tullio and Ronci (1997); Gaiotti et al. (1998); Gallo and Otranto (1998), and Favero and Spinelli (1999)]. However, unlike our work, studies for Italian economy on long sample does not test monetary or fiscal dominance using the sub-periods affected by potential structural changes.

Notwithstanding, the cointegration analysis might be biased by the presence of unattended structural breaks. Accounting for parameter shifts is crucial in cointegration analysis since this type of analysis normally involves long spans of data, which are more likely to be affected by structural breaks. In particular, our data covers 160 years of the history of the series, and during that period of time, the long-run relationship between primary budget deficit and public debt has probably changed due to alterations in monetary and fiscal policy, as well as reforms in the financial market. Thus, the information content of the basic model of the FTPL is subject to change over time, and all the empirical modelling studies that have not taken the possible changes and instabilities into account have likely failed to explain the variations in the relationship between the primary budget surplus and public debt. Therefore, as we argued before, it is very important to allow for structural breaks in our cointegration relationship.

**Table 6:** Tests for testing multiple structural breaks in cointegrated regression models from Kejriwal and Perron (2010)^a,b,c,d^

	Specifications^a^		
$y_{t}=\left\{ s_{t}\right\}$	$z_{t}=\left\{ 1,b_{t-1}\right\}$	$x_{t}=\left\{ \emptyset \right\}$	$M=2$
	$q=2$	$p=0$	$h=38$

	Tests^b^		
$\sup F_{T}(1)$	$\sup F_{T}(2)$	$UD\max$	
3.69	6.65$^{2}$	6.65	

	Number of breaks		
	Selected	Breaks	
		${\hat{T}}_{1}$	${\hat{T}}_{2}$
SP	0	–	–
LWZ	2	1910	1974
BIC	2	1910	1974

^a^$y_{t}$, $z_{t}$, *q*, *p*, *h*, and *M* denote the dependent variable, the regressors, the number of *I*(1) variables (and the intercept) allowed to change across regimes, the number of *I*(0) variables, the minimum number of observations in each segment, and the maximum number of breaks, respectively

^b^The null hypothesis of no structural changes (a stable cointegrating relationship) versus the alternative hypothesis of multiple structural changes

^c^Superscripts $^{1,2,3}$ indicate significance at the 10%, 5% and 1% levels, respectively

^d^The critical values are taken from Kejriwal and Perron (2010), Table 1.19 (critical values are available on Pierre Perron’s Web site), non-trending case with $q_{b}=1$

We next consider the tests for structural changes that are proposed in Kejriwal and Perron (2008, 2010). Given the span of the data, it seems unreasonable to expect the occurrence of one or more breaks. Since we have used a 20% trimming, the maximum numbers of breaks we may have under the alternative hypothesis is 3. Moreover, the intercept and the slope in Eq. (11) are permitted to change. Table 6 presents the results of the stability tests as well as the number of breaks selected by the sequential procedure (SP) and the BIC and LWZ proposed by Bai and Perron (2003). The $\sup F_{T}(2)$ test is significant at the 5% level, suggesting that the data do support a two-break model. The SP results do no suggest any instability, although the LWZ and BIC selects two breaks, which provides evidence against the stability of the long-run relationship. Overall, the results of the Kejriwal-Perron tests suggest a cointegrated model with two breaks estimated at 1910 and 1974 and three regimes, 1861–1909, 1910–1973 and 1974–2020.

Since the above reported stability tests also reject the null coefficient of stability when the regression is a spurious, we still need to confirm the presence of cointegration among the variables. With that end in mind, we use the residual based test of the null of cointegration against the alternative of cointegration with unknown multiple breaks proposed in Kejriwal (2008), ${\tilde{V}}_{k}({\hat{\lambda }})$.

**Table 7:** The residual based test of the null hypothesis of cointegration tests with multiple structural changes from Arai and Kurozumi (2007) and Kejriwal (2008)^a,b,c^

Two-breaks model				
Test ${\tilde{V}}_{2}({\hat{\lambda }})$	${\hat{\lambda }}_{1}$	${\hat{T}}_{1}$	${\hat{\lambda }}_{2}$	${\hat{T}}_{2}$
0.039$^{3}$	0.31	1910	0.71	1974

^a^The null hypothesis of cointegration with multiple structural changes versus the alternative hypothesis of no cointegration

^b^Superscripts $^{1,2,3}$ indicate significance at the 10%, 5% and 1% levels, respectively

^c^Critical values are obtained from simulations using 500 steps and 2000 replications. The Wiener processes are approximated by partial sums of *i*.*i*.*d*. *N*(0, 1) random variables

**Table:** 

Critical values	10%	5%	1%
${\tilde{V}}_{2}({\hat{\lambda }})$	0.066	0.082	0.117

Arai and Kurozumi (2007) show that the limit distribution of the test statistic, ${\tilde{V}}_{k}({\hat{\lambda }})$, depends only on the timing of the estimated break fraction ${\hat{\lambda }}$ and the number of I(1) regressors *m*.^3^ Since we are interested in the stability of the primary budget surplus and public debt coefficient, $\gamma$, we only consider model 3, which permits a slope shift as well as a level shift**.** Table 7 shows the results of the Arai-Kurozumi-Kejriwal cointegration tests allowing for two breaks. As before, the level of trimming used is 25%. As a result, we find that test ${\tilde{V}}_{2}({\hat{\lambda }})$ cannot reject the null of cointegration with two structural breaks at 1% level of significance. Therefore, we conclude that $s_{t}$ and $b_{t}$ are cointegrated with two structural changes estimated at 1910 and 1974.

To compare the coefficients obtained from the break models with those reported from models without any structural break, we estimate the cointegration Eq. (11) with a two-breaks model. The results with the sub-samples are presented in the last three columns of Table 5. The null of the deterministic cointegration between $s_{t}$ and $b_{t}$ is not rejected at the 1% level of significance in the three regimes.

For the case of the first (1861–1909) and third regimes (1974–2020) the estimated coefficients are positive and significantly different from zero at the 1% level, as in the full sample. Accordingly, public finances would also have been sustainable over the long-run and a Ricardian or MD regime, would have prevailed. Therefore, these sub-periods can not be characterized as one of fiscal dominance (non-Ricardian or FD regime) as suggest the FTPL theory. The exception would be the second regime (1910–1973), where the estimated coefficient is significantly different from zero but negative. In this case, fiscal policy would have been sustainable but a non-Ricardian or FD regime have prevailed as suggested the FTPL theory.

## Concluding remarks

In this paper, we provide a formal test of the sustainability of the Italian government deficit over the period 1861–2020. In an attempt to disentangle the implications of the Italian deficit on the interactions between fiscal and monetary policies, we have also analyzed the role played by monetary and fiscal dominance in order to get fiscal solvency along the period. The nature of that relationship would inform us about the dynamics of the successive Italian goverment’s macroeconomic performance along the time. Several studies have dealt with the issue of fiscal dominance in Italian economy using a short or a long sample, but no clear evidence on the prevalence of a MD or a FD regime was found. However, this type of evidence is not conclusive and it is not robust to the time span, as well to the estimation methodology.

Firstly, we provide a test for sustainability of the Italian government deficit over the period 1861–2020, using the fiscal theory of the price level (FTPL). This approach takes into account monetary and fiscal policy interactions and assumes that fiscal policy may determine the price level, even if monetary authorities pursue and inflation targeting strategy. To test empirically the prevalence of monetary dominance versus fiscal dominance and the sustainability of public finances in the basic FTPL equation, we have estimated a linear cointegrating relationship between the primary public surplus and the (lagged) level of public debt via a fiscal reaction function. For the full sample, we found that the estimated coefficient is positive and significantly different from zero at the 1% level. Accordingly, public finances would have been sustained over the long-run and a Ricardian or MD regime, as is suggested by the traditional macroeconomic approach, would have prevailed for the Italian economy. Therefore, the whole period can not be characterized as one of fiscal dominance (non-Ricardian or FD regime) as suggested the FTPL theory.

Secondly, the results of the Kejriwal-Perron tests suggest a cointegrated model with two breaks estimated at 1910 and 1974 and three regimes, 1861–1909, 1910–1973 and 1974–2020. Also, we find a deterministic cointegration between the primary public surplus and the public debt in the three regimes. For the case of the first (1861–1909) and third regimes (1974–2020) the estimated coefficients are positive as in the full sample. Hence, public finances would also have been sustainable over the long-run and a Ricardian or MD regime, would have prevailed. In particular, this result reveals that from the incorporation of Italy to the European monetary union in 1999 to date, the inflation targeting goal of the European single monetary policy has determined the price level in Italy. Therefore, these sub-periods cannot be characterized as one of fiscal dominance (non-Ricardian or FD regime) as suggested the FTPL theory. The exception would be the second regime (1910–1973), where the estimated coefficient is negative. In this case, fiscal policy would have been sustainable, but a non-Ricardian or FD regime was prevailing as suggested by the FTPL theory.

Lastly, we have analyzed the dynamics of the Italian public debt-to-GDP ratio is analysed during period 1861–2020. The longer than usual span of the data should allow us to obtain some more robust results than in most of previous analyses of long-run sustainability. We use recent procedures of testing for recurrent explosive behavior in order to identify episodes of explosive dynamic of public debt, which can be attributed to active budget (unsustainable) policies that ran in the past. We identify three episodes of explosive public debt behavior, which allows us to date-stamp their origination and termination, as well as the potential fiscal adjustments. The first episode of explosive behavior is dated in 1977–1994. The second episode, occurred in 1995–2007, was a fiscal adjustment. Finally, the third episode detected is a second period of explosive debt behavior (2008–2020).

The drop in economic activity together with the necessary policy responses to the pandemic sharply deteriorated the Italian government finances in 2020, challenging its sustainability in the medium and long term. Nevertheless, the government deficit and debt-to-GDP ratios have already started to fall in 2021 and are expected to continue declining as the economy recovers. As a result, these fiscal sustainability challenges are deemed to be constrained to the short term. However, considering the high level of government debt and the projected costs related to its ageing population, Italy’s fiscal sustainability challenges are still relevant for the medium and long term. In addition, new risks may emerge if the current accommodative monetary policy stance were to be reversed. In this context, prudent and effective management of government finances, both on the expenditure and the revenue side, as well as the effective implementation of the investments and reforms included in the recovery and resilience plan (RRP) to foster growth, remains crucial to better allocate public resources and achieve a sustainable fiscal adjustment.

As the European Commission suggests, the medium-term risks to fiscal sustainability are significant.^4^ First, the debt sustainability analysis shows that government debt is projected to rise from around 148% of GDP in 2022 to about 155% of GDP in 2032 in the baseline. This debt path is also sensitive to possible shocks to fiscal, macroeconomic and financial variables, as illustrated by alternative scenarios and stochastic simulations, all pointing to high risks. Moreover, the sustainability gap indicator S1 signals that an adjustment of the structural primary balance of 9.6 pps. of GDP would be needed to reduce debt to 60% of GDP in 15 years. Overall, the high risk reflects the current large deficit and high debt, the high sensitivity to adverse shocks, as well as the projected increase in public pension expenditure.

## Footnotes

1 We have checked the consistency between the both databases.

2 The critical values were obtained from simulations using 1,000 steps to approximate the Wiener process and 10,000 replications.

3 In our case, the critical values for the test are then simulated for the corresponding break fractions using 500 steps and 2000 replications. The Wiener processes are approximated by partial sums of *i*.*i*.*d*. *N*(0, 1) random variables.

4 See European Commission (2022).
